# Relationship between Organic Carbon and Opportunistic Pathogens in Simulated Glass Water Heaters

**DOI:** 10.3390/pathogens4020355

**Published:** 2015-06-09

**Authors:** Krista Williams, Amy Pruden, Joseph O. Falkinham, Marc Edwards

**Affiliations:** 1 Via Department of Civil and Environmental Engineering, Virginia Tech, 401 Durham Hall, Blacksburg, VA 24060, USA; E-Mail: kwilli@vt.edu; 2 Department of Biological Sciences, Virginia Tech, 5008 Derring Hall, Blacksburg, VA 24060, USA; E-Mail: jofiii@vt.edu

**Keywords:** opportunistic pathogens, premise plumbing, drinking water, *Legionella pneumophila*, *Mycobacterium avium*, *Pseudomonas aeruginosa*, *Vermamoeba vermiformis*, *Acanthamoeba*, assimilable organic carbon, biostability

## Abstract

Controlling organic carbon levels in municipal water has been hypothesized to limit downstream growth of bacteria and opportunistic pathogens in premise plumbing (OPPPs). Here, the relationships between influent organic carbon (0–15,000 µg ozonated fulvic acid /L) and the number of total bacteria [16S rRNA genes and heterotrophic plate counts (HPCs)] and a wide range of OPPPs (gene copy numbers of *Acanthamoeba polyphaga*, *Vermamoeba vermiformis*, *Legionella pneumophila*, and *Mycobacterium avium*) were examined in the bulk water of 120-mL simulated glass water heaters (SGWHs). The SGWHs were operated at 32–37 °C, which is representative of conditions encountered at the bottom of electric water heaters, with water changes of 80% three times per week to simulate low use. This design presented advantages of controlled and replicated (triplicate) conditions and avoided other potential limitations to OPPP growth in order to isolate the variable of organic carbon. Over seventeen months, strong correlations were observed between total organic carbon (TOC) and both 16S rRNA gene copy numbers and HPC counts (avg. R^2^ > 0.89). Although *M. avium* gene copies were occasionally correlated with TOC (avg. R^2^ = 0.82 to 0.97, for 2 out of 4 time points) and over a limited TOC range (0–1000 µg/L), no other correlations were identified between other OPPPs and added TOC. These results suggest that reducing organic carbon in distributed water is not adequate as a sole strategy for controlling OPPPs, although it may have promise in conjunction with other approaches.

## 1. Introduction

Although, *Legionella pneumophila* is the most frequently reported agent of waterborne disease outbreaks in the United States [1] other opportunistic pathogens such as *Mycobacterium avium*, *Acanthamoeba* spp., *Naegleria fowleri and Pseudomonas aeruginosa* are also increasingly recognized as public health concerns in potable water systems [2,3,4]. One key limitation in effectively introducing control measures for these pathogens is the relative lack of research on factors contributing to their growth and persistence under conditions representative of premise (*i.e.*, building) plumbing systems, which is the primary environment in which they are thought to replicate [5,6]. In contrast to traditional fecal pathogens, opportunistic pathogens in premise plumbing (OPPPs) tend to be resistant to disinfectant and subject to regrowth in the distribution system and premise plumbing. This calls for new paradigms aimed at shaping the microbiome of the premise plumbing rather than eradicating it [7]. For example, research is needed to determine how OPPPs might be controlled by manipulating various factors at the treatment plant, in the distribution system, and in premise plumbing. These include altering the source water chemistry, chemical/biological treatments, distribution system design, temperature control, dissolved oxygen levels, plumbing materials, and in-building disinfection strategies [8,9,10].

One logical approach in controlling pathogen regrowth involves limiting essential nutrients, such as organic carbon, from water flowing into potable water systems. In addition to generally limiting the regrowth of bacteria, this could also limit the growth of amoebae that graze on biofilms. Amoebae are of particular interest because they commonly serve as hosts for bacterial OPPPs and enhance their amplification [11]. Prior investigations have highlighted the promise of limiting organic carbon as a means of microorganism control in distributed water [12,13,14], but have focused almost exclusively on cold water and continuous flow conditions commonly encountered in water mains. This prior body of work revealed correlations between organic carbon nutrients [characterized as total organic carbon (TOC), assimilable organic carbon (AOC) or biodegradable organic carbon (BDOC)] and the regrowth potential of heterotrophic and coliform bacteria. Based on observations of higher microbial abundance above 50 µg AOC/L, this threshold has been discussed as a potential guideline for microbial control in disinfected systems [14,15]. A recent survey of eight unchlorinated water main distribution systems in the Netherlands further suggested that a more stringent AOC threshold of 5–10 µg carbon/L might offer additional benefit for controlling levels of *L. pneumophila* [10]. That report was consistent with an earlier study of two unchlorinated main distribution systems in which water with high total organic carbon (8 mg/L)\averaged about 10X higher levels of *Legionella spp.* when compared to a system with relatively low total organic carbon (<0.5 mg/L) [16].

Reducing organic carbon levels below certain thresholds (e.g., 5–10 µg carbon/L) could be an attractive approach for water utilities to protect public health. A potential advantage of such an approach is that it could offer broad reduction in health risks across all buildings served throughout a water system, even if disinfectants disappeared within premise plumbing [2]. While prior research indicates that limiting AOC is promising for *M. avium* and *L. pneumophila* control in cold continuously flowing water mains, it is critical to validate the effectiveness of this approach under conditions representative of premise plumbing water systems, where OPPPs may be more likely to replicate under the relatively warm, stagnant and chlorine-depleted conditions [15,17].

The research herein is the first to examine the relationship between the concentration of organic carbon in water and the numbers of relevant microorganisms, including three OPPPs (*L. pneumophila*, *M. avium*, and *Acanthamoeba* spp.) and relevant amoebae hosts (*Acanthamoeba* spp. and *V. vermiformis*), under conditions representative of premise-plumbing hot water systems. This was achieved using simulated glass water heaters (SGWHs), which provided a method of controlled and replicated long-term observations. This experimental approach was designed to isolate the effect of added AOC by eliminating potential leaching of organics that may occur from traditional premise plumbing materials and by maintaining temperature at the lower end of typical water heater operation (e.g., 32–37 °C) to ensure that temperature was optimal for bacterial growth. Further, long stagnation events (≥48 h) were employed to simulate low to moderate use, and no chlorine/chloramine residual was present to maximize the potential for microbial proliferation [5,18,19]. The SGWHs were operated over a broad range of total organic carbon (TOC) concentrations (0 to 15,000 μg/L) in the form of ozonated fulvic acid, a representative source of organic carbon in drinking water systems used in prior studies to demonstrate relationships between organic carbon and microbial regrowth [15].

## 2. Results

### 2.1. Correlations between Added Organic Carbon Concentration and 16S rRNA Gene Copies and HPC Counts

**Figure 1:**
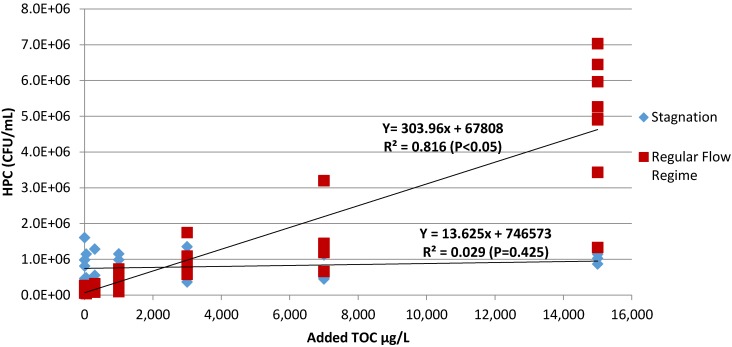
Effect of stagnation on HPC (CFU/mL) *vs.* TOC (Stagnation: 1X per week 80% water change—Experiment A3; Regular Flow Regime: 3X per week 80% water change—Experiment A2).

Strong correlations were consistently observed between TOC (0–15,000 µg/L) and both HPC counts (CFU) and total bacteria (as 16S rRNA gene copies) during intervals involving regular 3X per week 80% water changes in Experiment A1 (Average R^2^ values of 0.89 and 0.97 for 16S rRNA genes and HPCs, respectively) (Table 1). Experiment A2 was distinct from A1 in terms of accumulated biomass, a slightly lower temperature, and a longer stagnation during inoculation (25 *vs.* 13 days for A1). When 3X per week 80% water changes were resumed to initiate experiment A2, the correlations of HPCs and 16S rRNA to TOC correspondingly weakened (R^2^ = 0.65 and 0.63, respectively) (Table 1). Finally, in Experiment A3, when stagnation was extended by decreasing the 80% water change frequency to 1X per week, no significant correlations with added TOC were identified (Figure 1; Table 1).

**Table 1:** Experimental correlation summary: TOC (0–15,000 µg/L) *vs.* 16S rRNA genes, HPC.

Experiment Designator	Dates Performed	Average R^2^ Value 16S rRNA qPCR	Number of Time Points (n) 16S rRNA qPCR	Average R^2^ Value HPC	Number of Time Points (n) HPC
Preliminary Experiments	12/1/2009–4/15/2010	N/A	N/A	0.952	11
A1	4/28/2010–8/3/2010	0.971	3	0.889	9
A2	10/13/2010–3/7/2011	0.626	3	0.645	3*
A3	3/31/2011–4/18/2011	0.168	3	0.295	3

*Additional samples were collected but failed the QA/QC criteria.

### 2.2. M. avium

*M. avium* was occasionally correlated with added organic carbon, but only at lower levels of added TOC (0–1000 µg/L) (Experiment A2, Table 2). Colony counts confirmed the observed correlation for the one time point for which both data sets were available. However, correlations of *M. avium* with TOC were not observed consistently over the duration of the experiment. In addition, if higher levels of added TOC (>1000 µg/L) were included in the calculations, all correlations with respect to *M. avium* weakened substantially. No correlations between *M. avium* numbers and TOC concentration were identified in experiments A1 or A3, even at low TOC concentrations (0–1000 µg/L).

**Table 2:** Representative correlations of *M. avium* *vs*. TOC during regular flow regimes (Exp. A2).

	*M. avium* (R^2^ Value) qPCR	*M. avium* (R^2^ Value) Culture Enumeration
	Low Range TOC	Low Range TOC
	(0–1000 µg/L)	(0–1000 µg/L)
Day 2	0.007 (*p* = 0.890)	0.007 (*p* = 0.890)
Day 23	0.003 (*p* = 0.929)	0.004 (*p* = 0.929)
Day 51	0.819 (*p* = 0.034)	0.818 (*p* = 0.035)
Day 111	0.974 (*p* = 0.001)	N/A

*N/A indicates that the sample was not analyzed.

### 2.3. L. pneumophila

**Figure 2:**
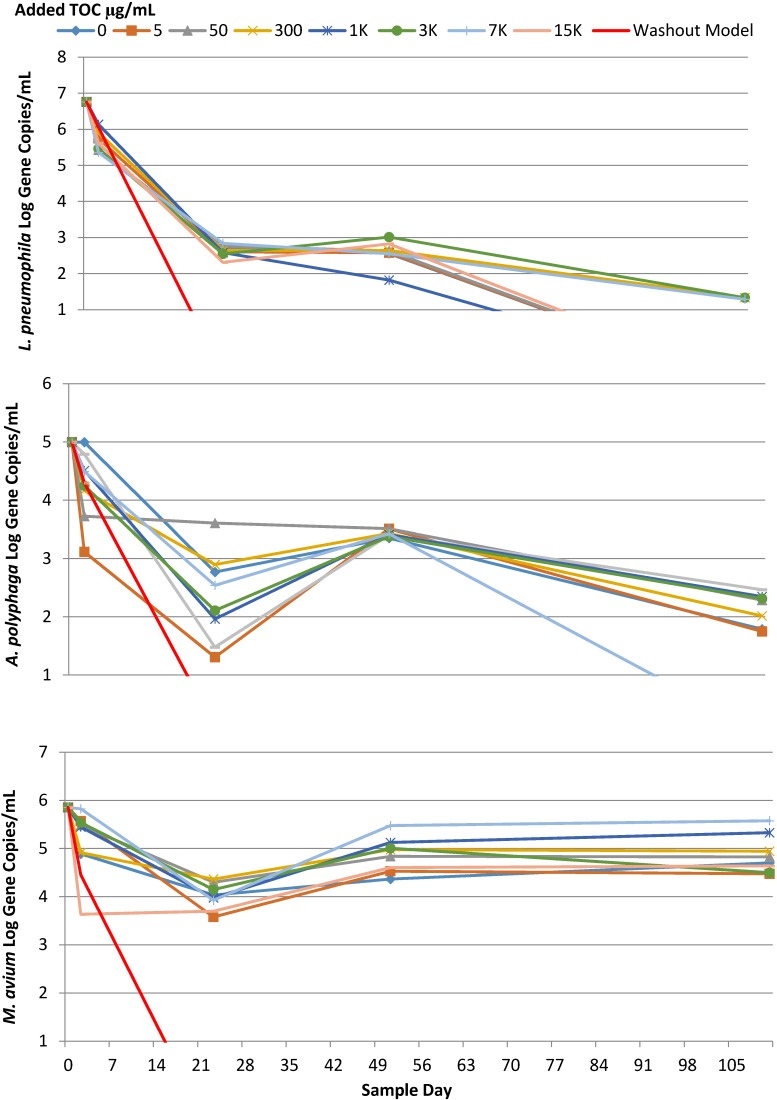
Comparison of (**Top**) *L. pneumophila*, (middle) *A. polyphaga*, and (**Bottom**) *M. avium* concentrations over time under a regular flow regime (3X per week 80% water change—Experiment A2) determined using qPCR.

*L. pneumophila* numbers did not correlate (either positively or negatively) with increasing TOC concentrations under any of the conditions investigated in this study. *L. pneumophila* from all SGWHs was measured in triplicate using qPCR for a total of 10 time points and the R^2^ with respect to added TOC ranged from −0.567 to 0.231 with a median R^2^ value of 0.174. Attempts were made to cultivate *L. pneumophila* for nine total time points in each of the three experiments and in no instances were colonies recovered. Despite the three variations in the experimental conditions that were expressly intended to induce the growth of *L. pneumophila*, added TOC was never found to be a driving factor for either *Legionella* amplification or persistence. In a typical experiment, concentrations of *L. pneumophila* initially present (>1.0 × 10^5^ gene copies/mL) decreased by several orders of magnitude as *L. pneumophila* cells and their DNA washed out from the SGWHs via the 80% water changes (Figure 2). A washout model, assuming that 20% of the original DNA remained in the SGWH after each water change, was plotted for comparison, which was in reasonable agreement with the overall trends considering that this model did not account for the retention of DNA in biofilms or the sustained release to effluent water. Overall, though *L. pneumophila* qPCR gene counts were frequently maintained above the washout model prediction, there was little evidence of increases above that expected from the initial inoculum.

### 2.4. A. Polyphaga

*A. polyphaga* was of interest in this study both as a host implicated with *L. pneumophila* as well as being an opportunistic pathogen in its own right. The results indicated no consistent correlations between low range TOC values (0–1000 µg/L) and *A. polyphaga* gene copy numbers over time (Experiment A2). While an increase in the numbers above the inoculum level was never observed under the conditions studied, there was significant evidence of persistence of *A. polyphaga* above washout predictions (Figure 2). Microscopic analysis revealed only encysted cells during the initial experimental phases, *i.e.*, during inoculation (17 September 2010) and Day 2 (13 October 2010), which may explain why *A. polyphaga* persisted without measureable growth. Occasionally, correlations were observed with lower levels of added organic matter (0–1000 µg/L, Table 3), but such correlations were transient.

**Table 3:** Representative correlation analysis of TOC *vs.* A. *polyphaga* (Experiment A2).

	*A. polyphaga* (R^2^ Value)
	Low Range TOC
	(0–1000 µg/L)
Day 2	0.991 (*p* = 0.004)
Day 23	−0.125 (*p* = 0.559)
Day 51	−0.118 (*p* = 0.569)
Day 111	0.683 (*p* = 0.084)

**Figure 3:**
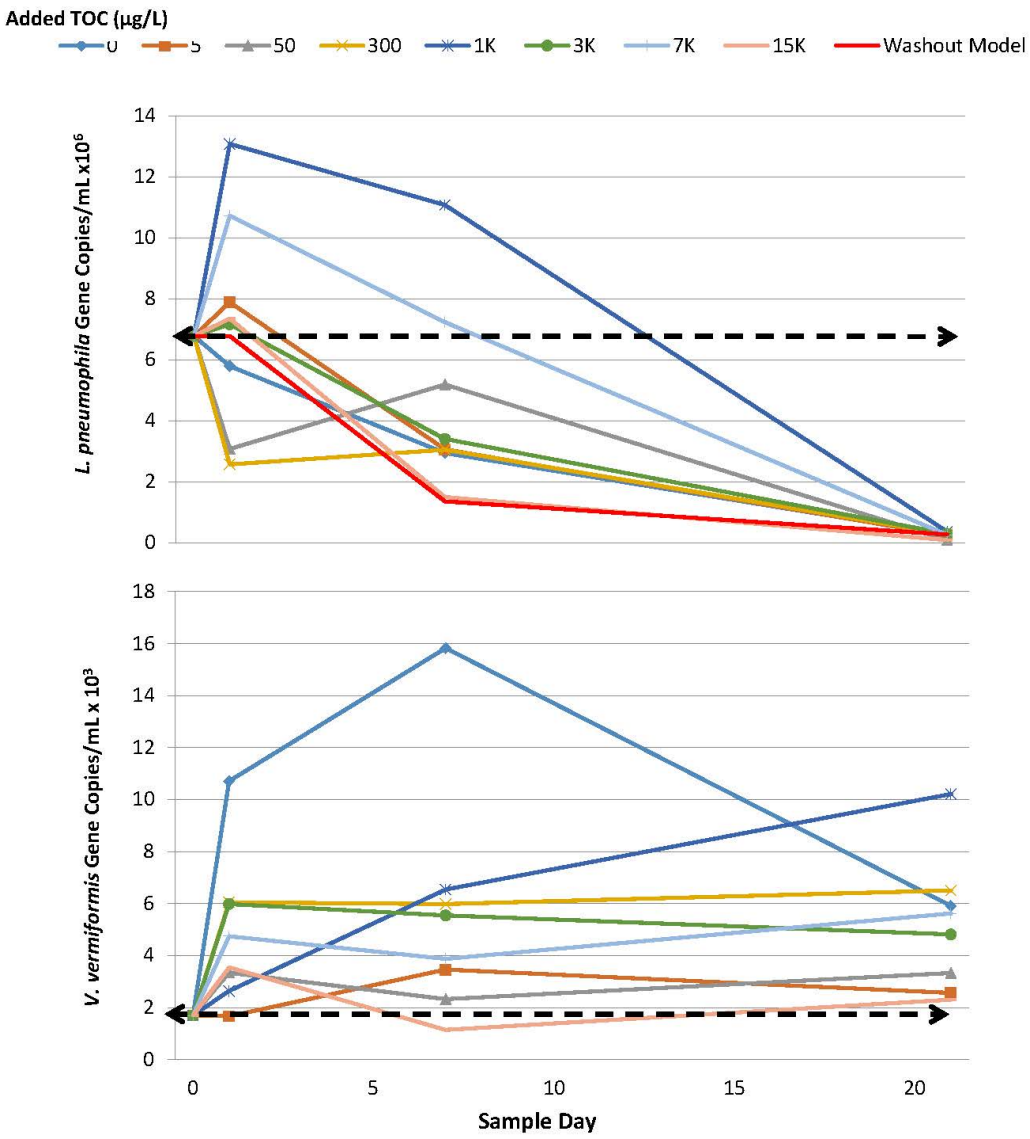
Comparison of *L. pneumophila* and *V. vermiformis* under 7-day stagnation (Experiment A3). The dashed line represents initial inoculated SGWH concentration.

As a result of the findings above, Experiment A3 modifications were implemented to further encourage *L. pneumophila* amplification. All SGWHs were inoculated with *V. vermiformis* at the beginning of Experiment A3 to provide an alternative host potentially better suited to the synthetic tap water. *V. vermiformis* was also found to have naturally colonized the A2 SGWHs at low concentrations upon the addition of GAC-treated Blacksburg, VA tap water. In the A3 Experiment, *V. vermiformis* rapidly increased in gene copy number by 1 log unit higher than the initial concentration (Figure 3). The concentrations peaked around two weeks after the inoculation of the SGWHs with no added TOC. Lower concentrations of added TOC appeared to encourage the growth of this protozoan as opposed to values above 5 µg/L. However, at no point were *V. vermiformis* numbers correlated with added organic matter (linearized plots of qPCR *vs.* added TOC resulted in R^2^ values of −0.175 (*p* = 0.862) on day 0, −0.462 (*p* = 0.004) on day 7, and −0.368 (*p* = 0.207) on day 21 after the start of Experiment A3).

Significant initial increases in *L. pneumophila* gene copies were measured by qPCR in Experiment A3 for a two-week period (>95% confidence), although no culturable *L. pneumophila* cells were detected over this same sampling interval (Figure 3). After the initial 2 weeks, the numbers of *L. pneumophila* declined in a manner that was closely approximated by the washout models. Notably, there was no correlation between *L. pneumophila* and added TOC, as the SGWHs with the greatest amplification contained moderate levels of added TOC (1000 and 7000 µg/L).

## 3. Discussion

Total bacterial numbers, as measured by 16S rRNA gene copy numbers and HPC colony counts, correlated with increasing organic carbon concentrations (0–1000 µg/L) with water changes three times per week, but the correlations were not significant when only considering lower levels of organic matter (<300 µg/L TOC, data not shown). For infrequent flow, such as the 1X weekly 80% water change encountered in Experiment A3, a general observation was that the strength of the correlation between the 16S rRNA gene copies and the HPC colony counts and organic matter weakened, even at very high levels of organic matter. This overall trend of a stronger correlation with relatively frequent water changes, and little or no correlation with long stagnation, may in part be a result of a shift to decay metabolism. Both endogenous and exogenous decay gained importance with longer stagnation intervals, reducing the overall likelihood of simple correlations between added TOC and either the 16S rRNA gene copies or the HPC colony counts. In previous work conducted by LeChevallier *et al.*, (2001), van der Kooij *et al.*, (2005) and Norton *et al.*, (2004) in cold water systems under continuous flow regimes, correlations were found between microbial numbers and AOC, and the identification of possible AOC thresholds (*i.e.*, <50 µg/L), where very effective control of heterotrophic (*i.e.*, HPC counts < 500 CFU/mL) and coliform bacteria could be achieved [14,15,20]. However, similar controls seem unlikely to be effective in premise plumbing hot water systems given that the very high levels of HPC colony counts (>10^6^ CFU/mL) and 16S rRNA gene copies were maintained even when no organic matter was added to the tap water synthesized from ultrapure water in which carbon was purposefully destroyed by UV. In retrospect, this is not surprising given the serious problems encountered with microbial re-growth (>10^4^ CFU/mL) observed in ultrapure water applications, for which levels of all nutrients were at practically achievable minima [21,22,23,24]. Common control strategies for controlling microbes in ultrapure water applications include avoiding stagnation and using periodic disinfection. Likewise, *P. aeruginosa* has been reported to proliferate in distilled water systems, and in very low AOC tap water [25,26]. In a recent survey in the Netherlands, others reported no relationship between biological stability of the distributed drinking water and percent sites positive for *P. aeruginosa* or *Stenotrophomonas maltophilia* in premise plumbing [27]. In this same study, *M. avium* was never observed, which was attributed to the water being unchlorinated and thus not selecting for disinfectant-resistant organisms.

The likelihood of correlations between microbial growth and AOC is also probably reduced in real‑world premise plumbing situations compared with the ideal situation tested herein using SGWHs. In practice, many premise plumbing systems are comprised of materials such as polyvinyl chloride, cross-linked polyethyelene (PEX), and various rubber materials that can potentially leach organic carbon into water and undermine the effectiveness of carbon limitations in the water after it enters buildings [5,6,17]. Inorganic materials, such as iron pipe, can also stimulate microbial growth, providing additional nutrients and generating AOC [28]. In addition, while more frequent flow regimes encountered in a typical building might strengthen the correlations between organic matter and overall bacterial concentrations, for waters with relatively high levels of secondary disinfectant, which are common in the U.S., the increased presence of disinfectant is likely to be the dominant factor reducing bacterial abundance [29].

*M. avium* numbers were sometimes strongly correlated with added organic matter levels below 1000 µg/L, which extends the observations of Norton *et al.*, (2004), obtained under conditions of continuous flow, to warm/hot water applications [15]. The failure of *M. avium* numbers to correlate with TOC above 1000 µg/L is consistent with the oligotrophic nature of *M. avium*, impairing its ability to compete with faster growing heterotrophic bacteria at higher levels of added TOC. A similar trend was reported for *P. aeruginosa*, for which the addition of organic carbon nutrients at a level of only 46 µg carbon/L reduced the levels of *P. aeruginosa* strain 6324 by ≈ 2 logs due to competition with other bacteria [25]. As a whole, the *M. avium* results confirm that competitive microbial ecology may interfere with any expectations for simple linear correlations between organic carbon or AOC with some pathogens in premise plumbing, which is consistent with the expectations based on emerging ‘probiotic’ concepts applied to potable water pathogen control [7].

For microorganisms such as *L. pneumophila*, *A. polyphaga*, and *V. vermiformis*, no definitive correlations between added TOC and bacterial abundance were observed during any timeframe or under any of the experimental conditions investigated. Indeed, although *L. pneumophila* and *A. polyphaga* persisted under some experimental conditions, there was limited evidence of amplification above the inoculum density. Correlations, when observed, were not consistent at either high (>1000 µg/L) or low (<1000 µg/L) concentrations of added TOC. In contrast to *M. avium*, *L. pneumophila* cannot directly utilize ozonated fulvic acids as a carbon source, making any relationship with TOC indirect, and thus undermining the potential effectiveness of a pathogen control strategy relying on organic matter removal or targeted levels of AOC/TOC. Further consideration of the complex predator-prey-parasite microbial ecology amongst the involved model organisms (*i.e.*, HPC, *A. polyphaga* and *V. vermiformis, L. pneumophila)* further complicates the viability of a pathogen control strategy based solely on organic carbon removal. The scope of this investigation was focused on monitoring the planktonic bulk water phase for the presence of OPPPs in an idealized model of a residential hot water heater. The planktonic phase (e.g., usage of tap and shower water) has particular relevance to water utilities as it is representative of where a consumer is most likely to come into contact with OPPPs. Based upon the design of this study, biofilm sampling would have proven disruptive to the native microbial ecology and may have compromised downstream data processing. However, biofilm sampling was attempted following preliminary investigations to investigate the potential of OPPPs residing in the biofilm, but failed to meet QA/QC criteria (data not shown). While biofilms certainly play a dynamic, complex role in providing a suitable medium for opportunistic pathogen growth and proliferation, sampling the localized microcosm was not necessary to meet the main objective of this investigation. Subsequent research on these same SGWHs indicated that *L. pneumophila* regrowth, under conditions where it does occur, is detectable and quantifiable in the bulk water phase [30]. Overall, while the results presented herein do suggest that levels of organic carbon in potable water can sometimes strongly impact premise plumbing microbial amplification, the factors at play in premise plumbing and the corresponding microbial ecology are complex, reducing the likelihood of the existence of simple relationships between pathogens and organic matter [2].

Interestingly, a transient increase in numbers of *L. pneumophila* in Experiment A3 did eventually occur. It is speculated that amongst other changes made in the experiment, a biodegradable growth source from dead cellular biomass may have played a role. A comparison between the 16S rRNA gene copy number analysis results and those from *L. pneumophila* indicated that the greatest *L. pneumophila* amplification occurred from a SGWH sample with 1000 µg/L of added TOC, for which *L. pneumophila* increased 6.0 × 10^6^ gene copies/mL compared with the inoculum value. This particular sample also had the greatest initial concentration of measured 16S rRNA gene copies/mL, *i.e.*, 8.6 × 10^7^. Considering that the particular SGWH had been stagnant for at least a two-week period prior to beginning formal 1X weekly 80% water changes, it is possible that the high copy number of 16S rRNA genes was present as dead cellular biomass. A recent study conducted by Temmerman *et al.*, (2006) found that *L. pneumophila* increased by 1.57 ± 0.32 log units through necrotrophic growth [31]. In this experiment, an increase in *L. pneumophila* of about 0.5 log units above the inoculation value was observed. These observations further underscore the complexity of *L. pneumophila* ecology in premise plumbing.

*L. pneumophila* marker genes were never detected in this completely synthetic tap water (e.g., experiment A1), suggesting that for unknown reasons, the synthesized water of Norton *et al.*, (2004) or the experimental conditions were somehow deficient for this pathogen [15]. A review of the literature revealed no other examples of such success under representative oligotrophic conditions (*i.e.*, organic carbon, phosphate, and nitrogen levels encountered in potable water) using completely synthesized potable water. The lack of a suitable synthesized water model for studying *L. pneumophila* growth under oligotrophic conditions is therefore a significant barrier to advancing the fundamental understanding of the driving factors for its amplification (and control) in drinking water systems [8].

As a final point, despite the lack of success for *L. pneumophila* growth in this work, a close inspection of prior literature further suggests that organic carbon limitation alone will not be adequate to control *Legionella* regrowth in premise plumbing. While van der Wielen and van der Kooij (2013) and Wullings *et al.*, (2011) reported encouraging trends linking lower levels of AOC to Legionella control in distributed mains water, the data presented in van der Kooij *et al.*, (2005) indicate that even water with a very low AOC at the point of entry (2–6 µg carbon/L) to a building supported amplification of *Legionella spp.* to 10^5^ CFU/mL in simulated premise plumbing systems with warm (25–35 °C) water and stagnation [10,16,20].

## 4. Experimental Section

To eliminate the potential of organic carbon leaching from materials, the SGWHs were comprised of 120-mL French square glass bottles (Qorpak, Bridgeville, PA) with polytetrafluoroethylene (PTFE) caps. Scaling down from a typical 40-gallon residential water heater resulted in a total calculated surface area to volume ratio increase from 0.05 cm^−1^ to 1.24 cm^−1^ for the SGWHs. Increased surface area in theory should further serve to support baseline conditions conducive to OPPPs regrowth. Prior to experimentation, all bottles were acid-washed, rinsed in reagent grade nanopure water and baked in a muffle furnace at 550 °C to combust residual organic carbon. SGWHs received synthesized potable water amended with 0, 5, 50, 300, 1000, 3000, 7000, or 15,000 µg/L as TOC from a stock solution of fulvic acid (ozonated to 50% reduction in overall UV_254_), which was originally isolated and purified from a freshwater lake using XAD (non-ionic solid sorbent) resin [32]. SGWHs were operated in triplicate at each target TOC level for a total of 24 SGWHs (8 levels of TOC X 3 replicates). The synthetic oligotrophic water recipe successfully used by Norton *et al.*, (2004) in a prior investigation of *M. avium* growth was utilized as baseline water for this experiment [15]. Ultimately, three variations in the baseline water (*i.e.*, 0 µg/L added TOC) were used in an attempt to increase *L. pneumophila* numbers as follows:

- **Water Source 1**: Nanopure water with UV carbon destruction and Norton *et al.* (2004) recipe to create synthesized tap water [15] (**Experiment: A1**).
- **Water Source 2**: A mixture of 90% Water Source 1 mixed with 10% Blacksburg tap water that had been circulated through a granular activated carbon (GAC) filter. (**Experiment A2**)
- **Water Source 3**: A mixture of 90% Water Source 1 mixed with 10% source water from Blacksburg tap water that had been circulated through a GAC filter. Additional trace nutrients of manganese (6 µg/L), iron (42 µg/L) and zinc (375 µg/L) were added along with a stock solution of trace amino acids, at levels encountered in potable water systems [33] (**Experiment A3**).

All three water sources were amended from frozen aliquots of stock fulvic acid (TOC of 1,240 mg/L), which were defrosted immediately prior to preparation of the SGWH influent. During operation, SGWHs were subject to regular 80% water changes three times weekly by decanting 80 mL, with minimal mixing or disturbance of any settled material, and replacing with the same volume of corresponding influent water in a biological safety cabinet under aseptic conditions.

### 4.1. First Phase of Testing: Experiment A1

SGWHs were filled to volume (100 mL) with the target TOC concentration (Water Source 1). Subsequently, 5 mL of water obtained from a local residential electric hot water heater was inoculated into each of the SGWHs to provide typical flora. The SGWHs were then incubated at 37 °C for 1 week. A 1-mL aliquot of *Acanthamoeba polyphaga* was added to each SGWH after an 80-mL (80%) water change, delivering a dose of 542 gene copies/mL from a culture of *Acanthamoeba polyphaga* strain ATCC 30871 grown in ATCC medium 2373 PYG. After an additional week of stagnation, a 1-mL aliquot of *M. avium* (5.84 × 10^4^ gene copies/mL) and *L. pneumophila* (5.09 × 10^9^ gene copies/mL) were inoculated with a culture of *M. avium* strain A5 grown in Middlebrook7H9 (M7H9) broth at 37 °C incubation for 7 days; *L. pneumophila* strain Philadelphia-1 (ATCC 33152) was grown in ATCC medium 1099 CYE at 37 °C for 7 days. Broth cultures were centrifuged (5000 × g for 20 min) and resuspended in the respective source water before inoculation into the SGWHs. *A. polyphaga* was grown to the stationary phase before inoculation to induce potential phagocytosis of heterotrophic and pathogenic microorganisms. *L. pneumophila* and *M. avium* were inoculated at the stationary phase, which is representative of microbial growth conditions in premise plumbing. Following inoculation, regular 80% water changes were commenced 3 times per week. The overall culturing/inoculation/acclimation approach was consistently applied across all experiments, with specific modifications noted in the following sections.

### 4.2. First Set of Changes to Baseline: Experiment A2

In experiment A2, Blacksburg, VA tap water was re-circulated through a GAC filter (3M^©^, Saint Paul, MN, USA) and incorporated into the baseline water at 10% of the total volume. The goal was to increase the likelihood of *L. pneumophila* growth by introducing inorganics, nutrients and microorganisms commonly found in potable water systems, with the acknowledged trade-off in terms of reduced experimental control. The temperature of the SGWHs was reduced from 37 to 32° C to idealize the conditions encountered at the bottom of electric water heaters, increase protozoan survival, and encourage cellular phagocytosis of *L. pneumophila*. Re-inoculation was completed using 1-mL aliquots as before but with modified concentrations: *A. polyphaga* (1 × 10^3^ amoeba/mL or 9.84 × 10^4^ gene copies/mL). SGWHs were allowed one week of stagnation at 32 °C before inoculation of *M. avium* (2.6 × 10^5^ CFU/mL or 7.15 × 10^5^ gene copies/mL) and *L. pneumophila* (3.2 × 10^3^ CFU/mL or 5.96 × 10^6^ gene copies/mL). Following an additional week of stagnation, routine water changes for the SGWHs were recommenced as performed previously.

### 4.3. Second Set of Changes to Baseline: Experiment A3

A second set of changes was made to the experimental conditions in a follow-up attempt to improve the ability of *L. pneumophila* to grow in the SGWHs. The surface area of the SGWHs was increased by 235.8 cm^2^ ± 5.89 cm^2^ through the addition of 1-mm diameter glass beads to encourage biofilm formation and increase biomass. The ambient pH was reduced from 8.6 to 7.5 via small additions of CO_2_ and the initial dissolved oxygen concentration was lowered to 4.0 mg/L to create a microaerophilic environment better suited to *L. pneumophila* according to a prior report [34]. In addition, a cocktail of amino acids known to serve as growth substrates for *L. pneumophila* was added to the medium [33]. Finally, *L. pneumophila* wild-type strains ATCC 33733, 33734, and 33823 isolated from potable tap water or shower samples were inoculated based on the hypothesis that they would be better adapted to survival in typical premise plumbing environments. The *L. pneumophila* cocktail was inoculated (6.7 × 10^4^ CFU/mL from plate counts and 6.78 × 10^6^ gene copies/mL). Furthermore, recent studies indicated that *V. vermiformis* may be a more relevant protozoan host for *L. pneumophila* and had naturally colonized the SGWHs during experiment A2 via the GAC filtered Blacksburg, VA water [11,35,36]. At the start of A3, additional *V. vermiformis* was inoculated into all SGWHs in this modified research phase (1.46 × 10^3^ amoeba/mL and 1.69 × 10^3^ gene copies/mL). Regular 80% water changes re-commenced following two weeks of stagnation and the water change frequency was reduced to a weekly basis.

**Table 4:** Summary of Experimental Details.

Experiment	Inoculation/Stagnation Periods	Dates Performed	Water Formulation; Temperature; Water Change Rate
**A1**	23 April 2010 Inoculation with 10 mL from Blacksburg hot water tank Incubate 1 week at 37 °C	5/18/10–8/3/10	Norton *et al.*, 2004; 37 °C; 3 water changes per week
	5 May 2010 Inoculation with *A. polyphaga* Incubate 1 week at 37 °C		
	12 May 2010 Inoculation with *L. pneumophila* and *M. avium* Incubate 1 week at 37 °C		
	18 May 2010 End stagnation period		
**A2**	17 September 2010 Inoculation with *A. polyphaga* Incubate 1 week at 37 °C	10/13/10–3/7/11 (5 months)	• 90% Norton *et al.*, 2004/ 10% GAC filtered Blacksburg, VA tap water; 32 °C; 3 water changes per week
	27 Septermber 2010 Inoculation with *L. pneumophila* and *M. avium* Incubate 1 week at 37 °C		
	13 October 2010 End stagnation period		
**A3**	18 March 2010 Inoculation of *V. vermiformis* Incubate at 37 °C	3/31/11–4/18/11 (3 weeks)	• 90% Norton *et al.*, 2004/10% GAC filtered Blacksburg, VA tap water with addition of amino acid, Mg, Fe, Zn and pH decrease to 7.5; 32 °C; 1 water change per week
	21 March 2010 Inoculation of *L. pneumophila* Incubate at 37 °C		
	31 March 2011 End stagnation period		

### 4.4. Nucleic Acid Extraction and Quantitative Polymerase Chain Reaction (qPCR) Deoxyribonucleic Acid (DNA) Extraction

Effluent water from triplicate SGWHs was combined into a single 240-mL sample at the time of the water change and was concentrated onto 47-mm diameter, 0.22-µm pore size mixed cellulose ester filters (Whatman, Piscataway, NJ) by vacuum filtration. The filters were fragmented using sterile tweezers and transferred to FastDNA^®^ SPIN Kit Lysing Matrix A tubes (MP Biomedicals, Solon, OH, USA). DNA was extracted as recommended by the manufacturer, except the FastPrep^®^ (MP Biomedicals, Solon, OH, USA) instrument was set at a speed of 4.0 for 20 seconds to reduce the possibility of DNA shearing. The resulting DNA was re-suspended in 100 µL of sterile purified water and preserved at −20 °C prior to analysis.

### 4.5. Quantitative Polymerase Chain Reaction (qPCR)

*L. pneumophila*, *V. vermiformis*, *Acanthamoeba* spp., *M. avium*, and total bacterial 16S rRNA genes were quantified as described and validated previously for drinking water samples by Wang *et al.*, (2012) [37]. All qPCR assays utilized a Bio-Rad CFX96 real time system (Hercules, CA) in a 20 μL total reaction volume. Negative controls consisting of template DNA replaced by molecular grade nanopure water and 10-fold serial dilutions of standard DNA (M13 PCR product of cloned targets) were included in triplicate in each qPCR assay and each sample was analyzed in triplicate.

### 4.6. Culture and Microscopic-Based Detection and Enumeration

#### 4.6.1. *L. pneumophila*

Each composite sample (0.1 mL) was diluted and spread directly on BCYE agar containing L-cysteine (2-Amino-3-sulfhydrylpropanoic acid). If overgrown, 1 mL of water sample was mixed with 1 mL of 0.2 M KCl-HCl solution and incubated at room temperature for 15 min (acid-treatment) before spreading. The plates were incubated at 37 °C and examined daily for *Legionella* colonies for up to 7 days prior to enumeration. Possible *Legionella* colonies were selected and streaked for isolation on BCYE agar lacking L-cysteine. If colonies were cultivated on plates lacking L-cysteine, the results were discarded as being non *Legionella* cells. Presumptive *Legionella* colonies were verified utilizing the respective qPCR assay as discussed above.

#### 4.6.2. *V. Vermiformis/A. Polyphaga*

For effluent water samples, 10 µL was added to a Petroff-Hauser Counting Chamber and amoeboid cells were counted at 100-magnification, noting evidence of trophozoite or cyst formation. The limit of detection for this assay is approximately 5 × 10^4^ amoeba/mL using the most probable number (MPN) methodology.

#### 4.6.3. *M. avium*

Each composite SGWH sample (0.1 mL) was diluted and spread directly on M7H10 agar containing 0.5% (vol/vol) glycerol and 10% (vol/vol) oleic-albumin (M7H10+GOA). Subsequently, the plates were inverted, incubated at 37 °C for up to 3 weeks, and subjected to colony enumeration.

#### 4.6.4. Heterotrophic Plate Counts (HPCs)

R2A agar (Difco) was utilized to enumerate HPC. Each composite SGWH sample (0.1 mL) was diluted and spread directly on R2A agar until dry. Subsequently, the plates were inverted and incubated at room temperature for one week before the colonies were counted [38].

### 4.7. Total Organic Carbon (TOC) and Biodegradable Dissolved Organic Carbon (BDOC) Measurement

A Sievers 800 TOC analyzer was used following Standard Method 5310A [38]. BDOC of the stock was analyzed utilizing the method of Servais *et al.*, (1987) with triplicate dilution of the fulvic acid stock solution to 3000, 7000, and 15,000 µg/L as TOC [39]. The samples were mixed with 10% V/V GAC filtered Blacksburg tap water to provide trace nutrients, inoculated with 1% V/V aliquots from the simulated water heater containing 1000 µg/L TOC and incubated at 32 °C. Dissolved organic carbon (DOC) was measured weekly over a 30-day period after passage through pre-rinsed 0.7-µm pore size glass fiber filters (Whatman, Piscataway, NJ, USA) to remove large aggregates and protozoan cells, wasting the first 10 mL of filtrate and collecting the next 10 mL for analysis. BDOC in the stock solution was measured based on the overall change in TOC during the 30-day incubation, indicating that the ozonated fulvic acid TOC stock consisted of 15% BDOC.

## 5. Conclusions

Although transient correlations sometimes existed between organic carbon and HPC colony counts or 16S rRNA gene copy numbers in SGWH effluents, correlations for *M. avium* were limited to lower levels of added organic carbon (< 1000 µg/L TOC) and no correlations were observed for *A. polyphaga*, *L. pneumophila*, or *V. vermiformis*. Longer stagnation events were associated with weakened correlations between organic carbon and either HPC colony counts or 16S rRNA gene copy numbers. At very low levels of added TOC (<300 µg/L), no correlations were observed between organic matter and HPC colony counts or 16S rRNA gene copy numbers, consistent with prior observations of very high microbial counts (>10^4^ CFU/mL) observed in ultrapure water application with long stagnation times and no disinfectant. Attempts to create model, oligotrophic synthetic drinking water that could support the growth of *L. pneumophila* were unsuccessful in this work. The absence of such a model is critical to identifying the key factors that drive *L. pneumophila* persistence and growth in drinking water systems and premise plumbing. Overall, simple relationships between these opportunistic pathogens and organic carbon in the water were not observed, due to the relatively complex microbial ecology of premise plumbing pathogens.
